# Filamentation of *Campylobacter* in broth cultures

**DOI:** 10.3389/fmicb.2015.00657

**Published:** 2015-06-30

**Authors:** Nacheervan M. Ghaffar, Phillippa L. Connerton, Ian F. Connerton

**Affiliations:** Division of Food Sciences, School of Biosciences, University of Nottingham, LoughboroughUK

**Keywords:** *Campylobacter*, filamentation, morphological changes, morphotypes, survival, intracellular ATP

## Abstract

The transition from rod to filamentous cell morphology has been identified as a response to stressful conditions in many bacterial species and has been ascribed to confer certain survival advantages. Filamentation of *Campylobacter jejuni* was demonstrated to occur spontaneously on entry in to stationary phase distinguishing it from many other bacteria where a reduction in size is more common. The aim of this study was to investigate the cues that give rise to filamentation of *C. jejuni* and *C. coli* and gain insights into the process. Using minimal medium, augmentation of filamentation occurred and it was observed that this morphological change was wide spread amongst *C. jejuni* strains tested but was not universal in *C. coli* strains. Filamentation did not appear to be due to release of diffusible molecules, toxic metabolites, or be in response to oxidative stress in the medium. Separated filaments exhibited greater intracellular ATP contents (2.66 to 17.4 fg) than spiral forms (0.99 to 1.7 fg) and showed enhanced survival in water at 4 and 37°C compared to spiral cells. These observations support the conclusion that the filaments are adapted to survive extra-intestinal environments. Differences in cell morphology and physiology need to be considered in the context of the design of experimental studies and the methods adopted for the isolation of campylobacters from food, clinical, and environmental sources.

## Introduction

*Campylobacter* is frequently responsible for foodborne bacterial gastroenteritis worldwide (World Health Organization [WHO], 2013). *Campylobacter* cells are usually slender, spiral shaped rods measuring 0.2–0.8 μm wide and 0.5–5 μm in length (Vandamme, 2000) but like many other microorganisms, filamentous forms have been observed under certain circumstances (Griffiths, 1993; Thomas et al., 1999; Apel et al., 2012; Cameron et al., 2012). Filamentation has been identified in many different bacteria and is thought to occur through inhibition of cell division, metabolic changes, or DNA damage which includes the SOS response resulting in the inhibition of septum formation whilst the chromosome is repaired (Justice et al., 2008). It has frequently been associated with stress and starvation conditions during which it may confer survival advantages (Justice et al., 2008). Moreover, it has been suggested that filamentation could represent a programmed response to unfavorable environments that aids the bacterium’s survival (Justice et al., 2008) and may enhance virulence (Mulvey et al., 2001; Stackhouse et al., 2012). Alternatively, filamentation may simply occur through an inadvertent loss of control of the normal cell division process. Whichever scenario is true may depend on the species of bacteria and the type of environmental stress encountered. Examples of environmental signals that have been identified to induce filamentation include: starvation, exposure to oxidative stress, reduced water activity, the presence of quorum sensing molecules, antibiotics, or host immune effectors (Allison et al., 1992; Jones et al., 1996; Janion, 2001; Miller et al., 2004). Filamentation as a response to sublethal stress has been observed in a number of foodborne bacteria, which have led to concerns that these bacteria may rapidly divide once the growth conditions become permissive to cause spoilage or disease (Jones et al., 2013).

Filamentation in *Campylobacter* has also been observed in response to mutation of the response regulator RacR and its sensor RacS, which are involved in the heat shock response (Apel et al., 2012), in response to treatment with certain antibiotics such as sitafloxacin (Yabe et al., 2010) and as a general response to hyperosmotic stress (Cameron et al., 2012). Importantly, for *Campylobacter jejuni* and *Helicobacter pylori* broth cultures, grown in a microaerobic atmosphere, filamentation occurred spontaneously on entry in to stationary phase (Griffiths, 1993; Fawcett et al., 1999; Thomas et al., 1999; Wright et al., 2009). Here nutrients may become depleted, potentially leading to starvation stress or there may be a buildup of metabolites present in the spent medium but the lack of a specific stress trigger distinguishes filamentation of these two related bacterial genera from other bacteria where stationary phase cells are generally reduced in size (Nyström, 2004). Moreover, elongated cells can be readily identified in scanning electron micrographs of *Campylobacter* biofilms (for examples see Kalmokoff et al., 2006; Brown et al., 2014) indicating filamentation may occur naturally in situations where biofilms form.

The aim of this study was to investigate the cues that give rise to filamentation of *C. jejuni* growing in broth cultures. We also aimed to investigate the viability of the individual component cells of the filament using vital staining, determine any strain dependency and any possible differences in the ability of the two different morphotypes to survive unfavorable conditions.

## Materials and Methods

### Bacterial Strains

*Campylobacter* strains that were used for this study included: HPC5, HF5, (*C. jejuni* poultry isolates); NCTC11168, NCTC12661 (35925B2), 81-176, PT14, 81116 (*C. jejuni* reference strains isolated from humans); OR4451C, OR5482C (*C. coli* poultry isolates), and FB1 (*C. coli* human isolate). All strains were stored at-80°C in Microbank vials (Pro-Lab Diagnostics, Wirral, UK).

### Growth in Liquid Cultures

Nutrient Broth Number 2 (NB2; Oxoid, Basingstoke, UK) and Mueller Hinton Broth (MH; Oxoid) were prepared according to manufacturer’s instructions. MEM (minimum essential medium) without glutamine and phenol red (Catalog number 51200-038; Life Technologies Ltd, Paisley, UK), with and without addition of 10 mM sodium pyruvate (Sigma Aldrich, Gillingham, UK), as an energy source were also tested. The *Campylobacter* inoculum was prepared by making a suspension containing approximately 10^8^ CFU/ml from an overnight culture grown on blood agar (BA; Oxoid) containing 5% (v/v) of defibrinated horse blood (TCS, Buckingham, UK) at 37°C incubated under microaerobic conditions (approximately 7% O_2_, v/v) obtained by the evacuation/replacement technique (Bolton and Coates, 1983). The replacement gas mix contained (5% v/v H_2_, 10% v/v CO_2_, and 85% N_2_). To prepare growth curves, 0.1 ml of the *Campylobacter* inoculum was added to three individual 250 ml conical flasks containing 50 ml of medium and the flasks placed in anaerobic jars (Oxoid) under microaeobic conditions generated as described above. The jars were placed in an orbital shaker and shaken at 100 rpm at 37°C with sampling at appropriate intervals. For each time point, an aliquot was serially diluted in maximal recovery diluent (MRD; Oxoid) and the microaerobic atmosphere re-generated. Enumeration of campylobacters was carried out by the Miles and Misra method on *Campylobacter* blood-free selective agar plates without supplement (CCDA; Oxoid) and incubated microaerobically at 42°C for 48 h. The morphology of the cells was examined microscopically over 10 independent fields using bright field, epifluorescence and Gram-stain for each time point. Pre-used NB2 was prepared by carrying out the above procedure with incubation for 48 h and confirmation of the formation of filamentous cells. The cell growth was removed by centrifugation at 13,000 *g* for 15 min, and the supernatant filtered through a 0.2 μm filter (cellulose acetate, Sartorius Stedim Biotech, Epsom, UK). The pre-used filter sterilized medium was inoculated as described above to prepare growth curves. To reduce the potential accumulation of free radicals in MEM with pyruvate, the medium was supplemented with 0.15% w/v starch (Sigma Aldrich) prior to sterilization. Statistical differences were assessed by ANOVA from the Excel Data Analysis package (Microsoft Corporation, Redmond, WA, USA).

### Fluorescent Cell Staining (Syto9/Propidium Iodide)

Bacterial suspensions (1 ml) were mixed with 1.5 μl of Syto 9 (absorption/emission 485/498 nm) and 1.5 μl propidium iodide (absorption/emission 535/617 nm) from LIVE/DEAD^®^ BacLight^TM^ Bacterial Viability Kit (Life Technologies) and incubated in the dark for 20 min at room temperature. A 5 μl aliquot was applied to the center of a clean glass microscope slide. An 18 mm^2^ coverslip was placed over the suspension. The slides were examined over 10 min at a magnification of 1,250 (100×, plan Apo) with an epifluorescence microscope (Labophot; Nikon, Tokyo, Japan) and images captured from 10 independent fields using a vertical mounted digital camera.

### Separation of Short Spiral and Filamentous Morphotypes

In order to separate the short spiral and filamentous forms, three independent biological replicates of *C*. *jejuni* 12661 or PT14 were inoculated into 500 ml of sterile MEM with sodium pyruvate, to a final density of approximately 10^5^ CFU/ml. The flasks were incubated in a shaking incubator at 100 rpm, at 37°C under microaerobic conditions for 30 h. The short spiral cells were separated from filamentous cells by collecting the filtrate from the culture that was passed through a 0.8 μm sterile nitrocellulose membrane (Sigma–Aldrich). The filtration was repeated with a fresh filter. The filamentous forms were obtained by flushing the first filter with fresh MEM (with pyruvate) and collecting the eluate. The suspensions were examined microscopically and diluted to contain approximately 5 × 10^6^ CFU/ml in either sterile water (reverse osmosis) or NB2 to examine their survival characteristics.

### Comparison of the Survival of the Short Spiral and Filamentous Morphotypes

Suspensions of the separated morphotypes in either water or NB2 (prepared as described above) were either incubated at 37 or 4°C for 96 h under microaerobic conditions. Viable counts were performed as described above at 24 h intervals.

### Determination of Intracellular ATP

The ATP concentrations were measured by luciferase luminescence using a commercial kit according to the manufacturer’s instructions (Promega, Southampton, UK). Either suspensions of selected morphotypes (prepared as above) or culture suspensions collected at various time points were centrifuged at 13,0000 *g* for 15 min and the cell pellets washed in 1ml of TA buffer (20 mM Tris-acetate buffer pH 7.75) before re-pelleting. The cell suspensions were lysed with 1% (w/v) trichloroacetic acid in TA buffer. The ATP assays were performed by adding 10 μl of the cell extract to a polypropylene tube containing 100 μl of recombinant luciferase/luciferin reagent, followed by gentle mixing and immediate reading in a pre-blanked luminometer (Turner Designs TD 20e, Promega). The signal was integrated over 10 s with a 2 s delay, and reported in relative light units (RLU) that could be converted to ATP concentrations using a pre-prepared standard curve. ATP contents were normalized according to either viable counts or microscopic cell counts or protein content (Bradford reagent; Pierce).

## Results

### Confirmation of Filamentation on Entry to Stationary Phase and the Imaging of Filament Component Cells by Vital Staining

**FIGURE 1:**
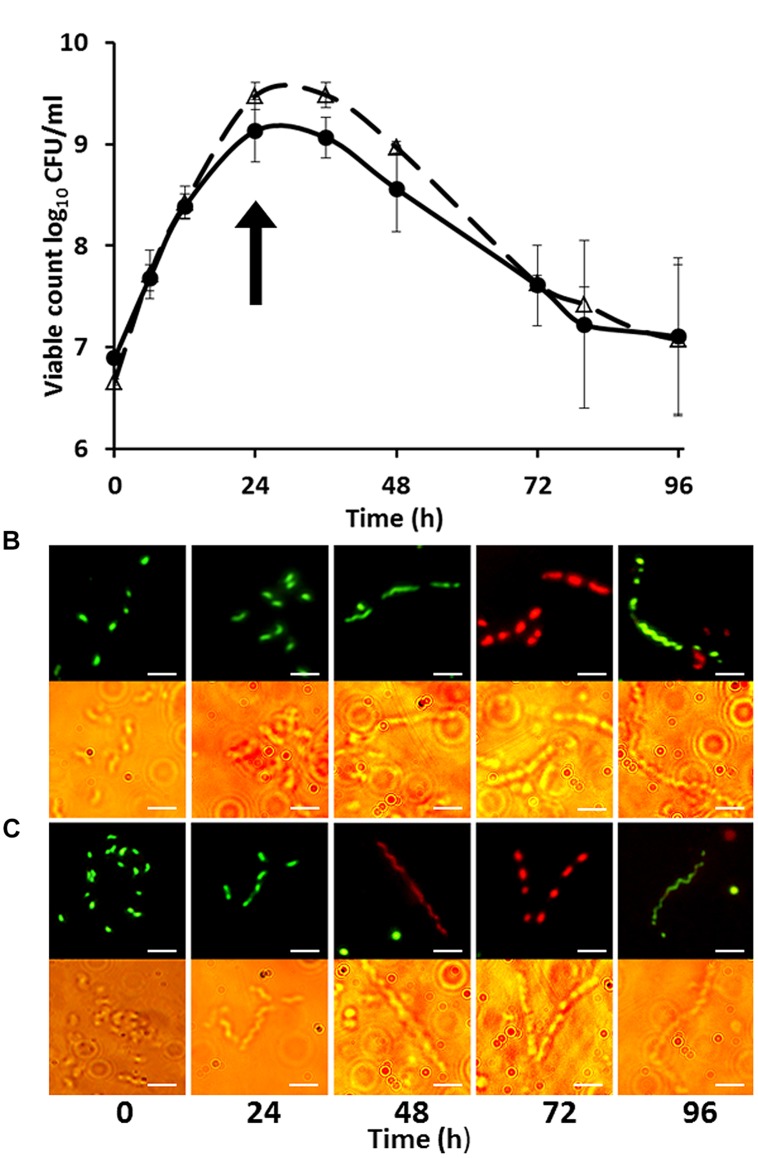
**Growth curves **(A)** of *Campylobacter jejuni* strains HPC5 (△) and HF5 (●) and morphological changes during growth of *C. jejuni* HPC5 **(B)**, and HF5 **(C)** in NB2.** Arrow in **(A)** indicates the time point at which the filamentous cells were first observed (on entry to stationary phase). Error bars are ±SD for *n* = 3. In **(B)** and **(C)** the upper row shows cells stained with fluorescent stains whilst the lower row shows bright field microscopy image of the same cells. Scale bar, 5 μm.

Growth curves and microscopic images of *C. jejuni* strains HPC5 and HF5 growing in NB2 over 48 h are shown in **Figures 1A–C.** These data confirm previous observations that on entry to stationary phase, after 24 h, progressively longer filaments are formed (Griffiths, 1993). The morphological changes coincided with different phases of growth. Exponentially growing cells showed typical short spiral forms (1.5–2.5 μm), while mid-stationary phase cells had become elongated. Cell populations in decline phase featured long filaments and the appearance of coccal forms. Vital staining was carried out using a combination of Syto 9, which stains intact cells green and propidium iodide, which only penetrates membrane damaged cells, staining them red. This did not produce the expected result, with many motile and therefore essentially live red-stained cells, appearing in the exponentially growing population. It appears that propidium iodide was able to enter live cells and was not a good indicator of viability for *Campylobacter*. Vital staining of the stationary phase cells showing filamentation was variable, with the cell filaments, either predominantly red (staining with PI) or predominantly green (stained with Syto-9) with relatively few cells containing both fluors (integrated images colored yellow). Interestingly comparison of the bright field and fluorescent microscope images presented in **Figures 1B,C**, clearly showed that the filaments contained cells that were unstained between the stained cells, and remarkably these often appeared in a regular interspersed pattern as demonstrated at the 72 h time points for *C. jejuni* strains HPC5 and HF5 in **Figure 1**.

### Growth of *Campylobacter* in Pre-Used Medium to Investigate the Potential Role of Quorum Sensing, Depletion of Nutrients

**FIGURE 2:**
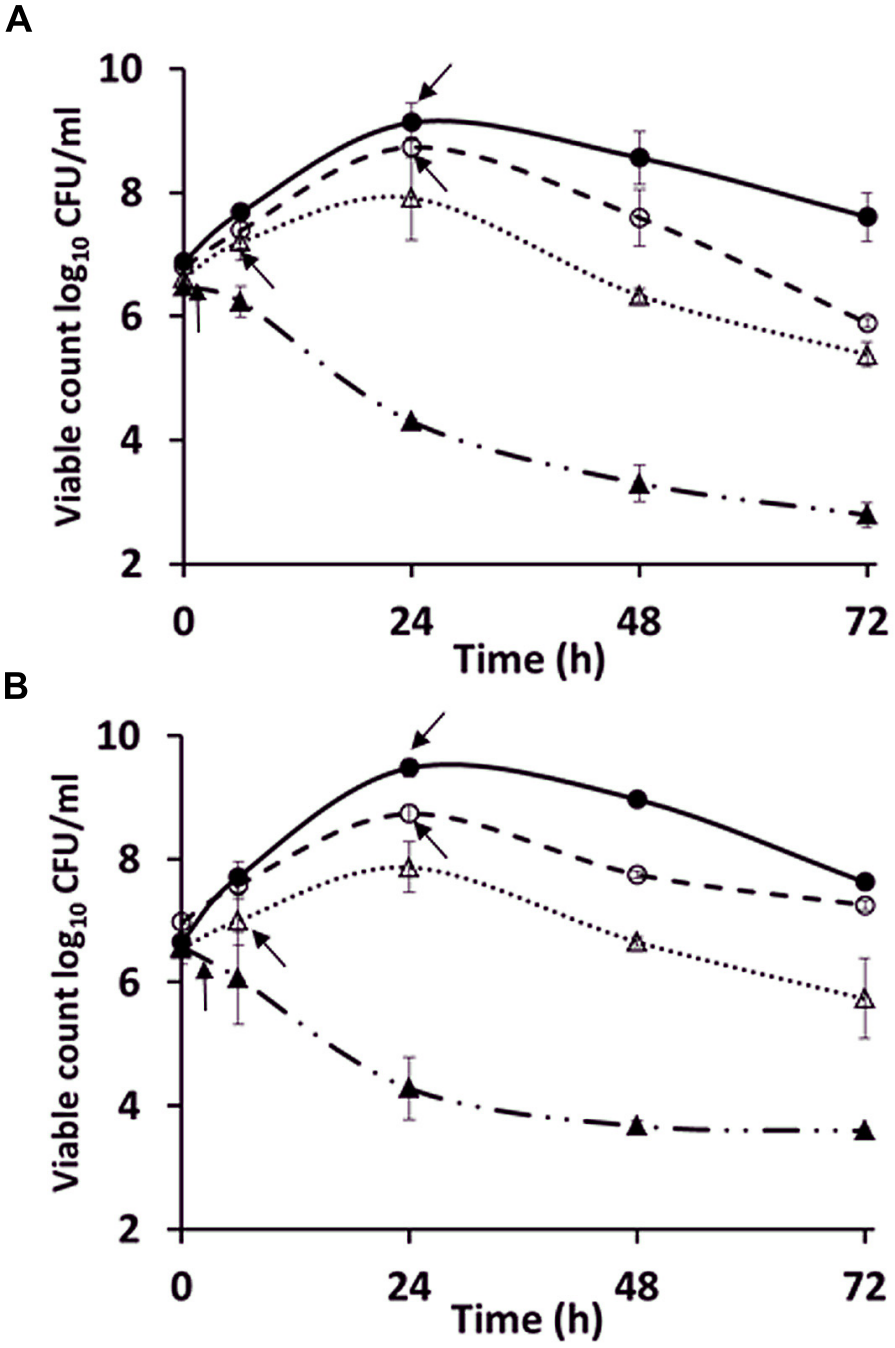
**Comparison of the growth of *C. jejuni* strains **(A)** HPC5 and **(B)** HF5 in: NB2 (●), pre-used NB2 (○), minimum essential medium (MEM; ▲), and MEM supplemented with 10 mM sodium pyruvate (△).** Arrows indicate the time points at which filamentous cells were first observed. Error bars are ±SD for *n* = 3.

The exponential growth rates of *C. jejuni* HF5 and HPC5 in the pre-used NB2 medium (*k* = 0.14 /h for both strains), were marginally reduced compared to fresh medium (*k* = 0.18 /h for HF5 and *k* = 0.25 /h for HPC5; **Figure 2**). This difference was significant for HPC5 (*p* = 0.03; ANOVA) but not significant for HF5 (*p* = 0.4; ANOVA). Although not optimum, the pre-used medium was able support exponential growth, suggesting that essential nutrients had not become significantly depleted in the course of achieving stationary phase in the previous culture. Moreover the growth period required for filamentation to occur were similar for fresh and pre-used NB2, suggesting that the campylobacters were not responding to the release of soluble signal molecules as observed for auto inducer molecules involved in quorum sensing.

**FIGURE 3:**
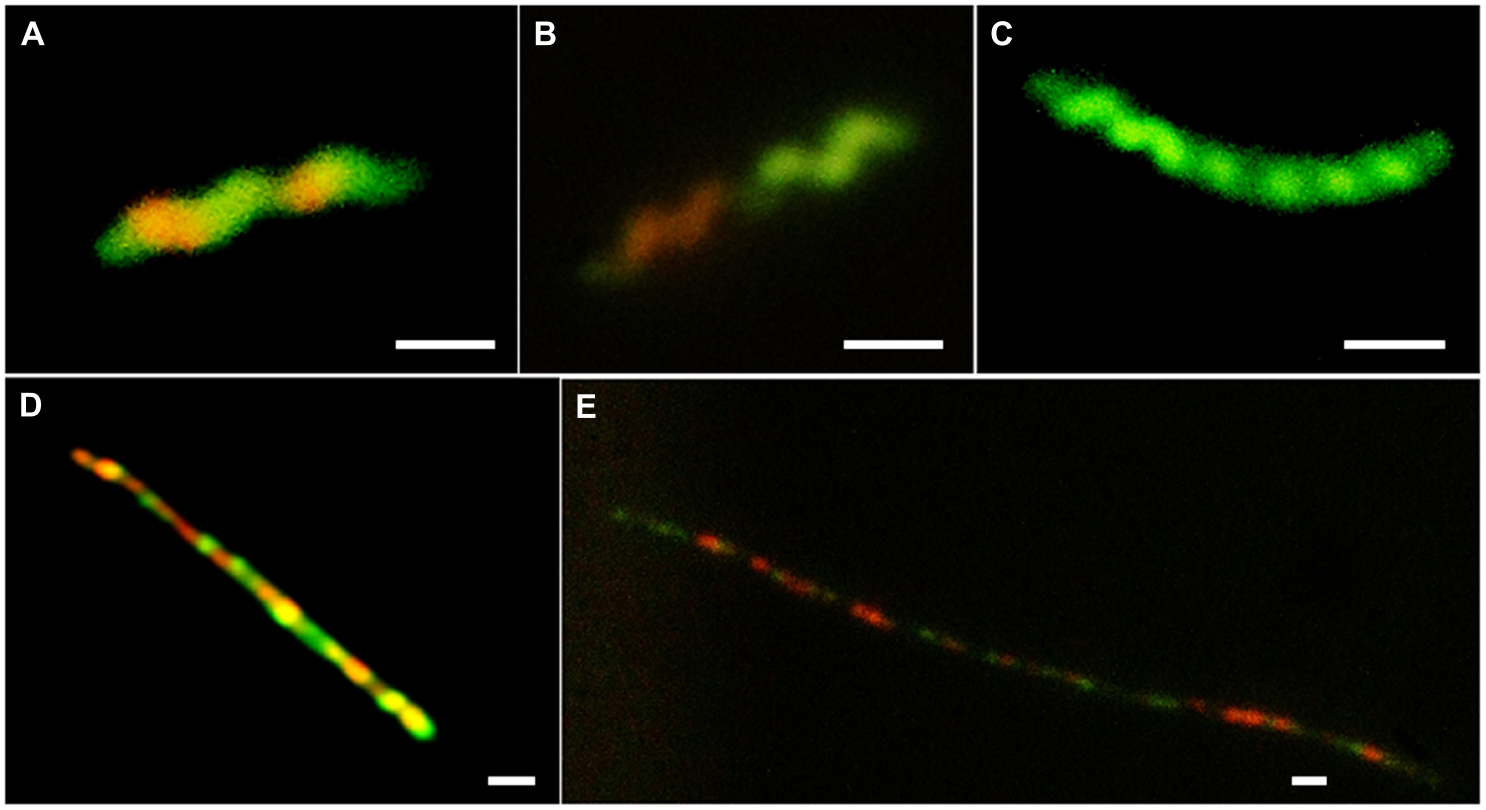
**Examples of variation in vital staining observed during growth in MEM and in MEM supplemented with 10 mM sodium pyruvate; **(A)***C. jejuni* HPC5 after 2 h incubation in MEM, **(B)***C. jejuni* HPC5 after 4 h incubation in MEM, **(C)***C. jejuni* HPC5 after 6 h incubation in MEM; **(D)***C. jejuni* 12661 after 48 h incubation in MEM supplemented with 10 mM sodium pyruvate; and **(E)***C. jejuni* 12661 after 72 h incubation in MEM supplemented with 10 mM sodium pyruvate**. Scale bar 1 μm.

To investigate further the role of nutritional limitation as a possible cause of filamentation, growth curves were prepared using MEM for *C. jejuni* HF5 and HPC5. Under these conditions filamentation was observed as early as 2 h (**Figure 2**; indicated by arrows), but with no increase in viable count and no filaments greater than 10 μm in length (**Figures 3A–C**). Some individual cells were stained both red and green (**Figure 3A**). Addition of sodium pyruvate, as an energy source, produced an increase in the viable count and filamentation was delayed such that filaments appeared after 4–6 h of incubation. Longer incubation periods produced filaments greater than 10 μm in length, which stained with both Syto 9 and PI. This dual staining was particularly evident in a third strain investigated, *C. jejuni* 12661 (**Figures 3D,E**).

### Growth of *Campylobacter* in Starch-Containing Media to Reduce Oxidative Stress

To assess if the provision of starch as an antioxidant, in the culture media could prevent or delay the onset of filamentation, we cultured campylobacters in either Mueller Hinton broth or MEM (with pyruvate) containing starch. Neither of these alternative growth media altered the time of entry to stationary phase or the subsequent observation of filamentous morphotypes (results not shown). The accumulation of growth related oxidative stressors in the culture medium was probably not a causal effect of filamentation.

### Filamentation in Different Strains of *C. jejuni* and *C. coli*

To determine if filamention was widespread amongst *C. jejuni* and *C. coli* a larger group of strains was examined. These were grown in MEM with sodium pyruvate and morphological changes compared in terms of the time taken for filamentation to be first observed (**Table 1**). Differences were observed, but filamentation was a feature of all the strains tested (cells > 5 μm) apart from *C. coli* strain (FB1), which did not form filaments, the viable count did not increase, and this strain produced coccal forms as early as 6 h under these conditions. One strain, *C. jejuni* 12661, produced particularly long filaments and was therefore chosen for further experiments involving separation of filaments from other morphotypes.

**Table 1:** Viability and filamentation of *Campylobacter* strains growing in different media.

Media	Strain	Time to achieve stationary phase (h)^*^	Time to first filaments (h)^†^	Viable count at first filamentation (CFU/ml)
NB2	HPC5	30	24	3.0 × 10^9^
	HF5	30	24	1.3 × 10^9^
Pre-used filtered NB2	HPC5 HF5	24 24	24 24	2.5 × 10^9^ 8.3 × 10^8^
Minimum essential medium ({MEM; (with sodium pyruvate)	HPC5	24	6	1.0 × 10^7^
	HF5	24	6	1.6 × 10^7^
	FB1^$^ OR4451C^$^ OR5482C^$^	24 24 24	24^‡^ 6 6	4.0 × 10^6^ 3.32 × 10^7^ 6.0 × 10^7^
	81116	24	6	6.2 × 10^7^
	11168	24	6	1.7 × 10^7^
	12661	24	6	2.1 × 10^7^
	81-176	24	6	1.3 × 10^7^
	PT14	24	6	5.2 × 10^7^
MEM (without sodium pyruvate)	HPC5 HF5	- –	2 2	2.6 × 10^6^ 1.6 × 10^6^

*Determined from growth curve inoculated with ~10^6^ CFU.

*^†^Determined microscopically, ^‡^Double length cells, ^$^Campylobacter coli.*

### Filamentous Forms Show Increased Survival in Water

**FIGURE 4:**
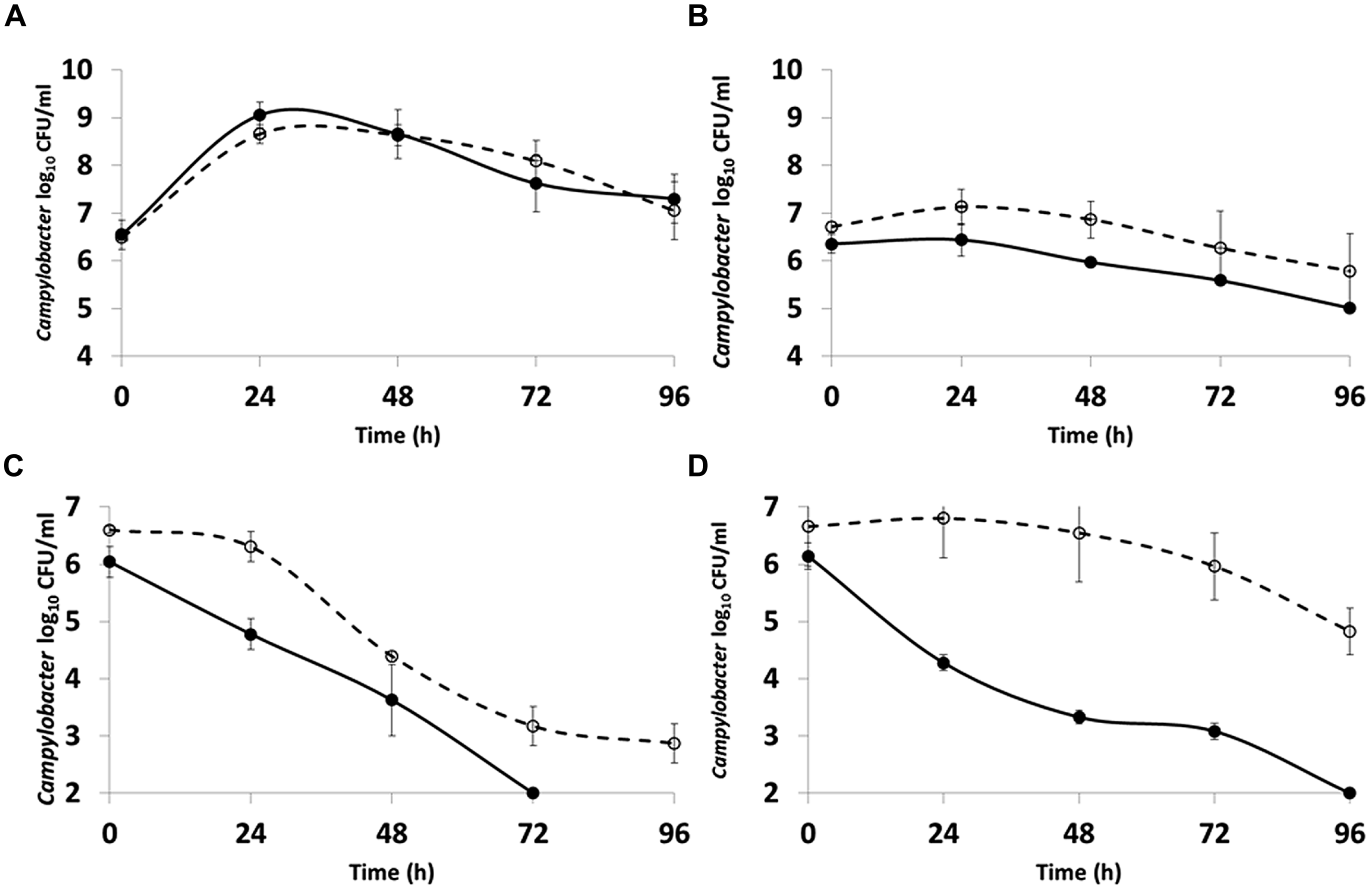
**Comparison of the survival of *C. jejuni* 12661 morphotypes incubated microaerobically at: **(A)** 37°C in NB2; **(B)** 4°C in NB2; **(C)** 37°C in water; **(D)** 4°C in water.** Short spiral forms (●), filamentous forms (○). Error bars are ±SD for *n* = 3.

In order to compare the characteristics of the filamentous and spiral cell forms coexisting in stationary phase cultures of *C. jejuni* 12661 (exaggerated filamentation phenotype) were separated using membrane filtration. A 0.8 μm filter prevented the passage of long filamentous forms and did not hinder the passage of short spiral forms present after 30 h incubation in MEM with sodium pyruvate at 37°C under microaerobic conditions. The survival characteristics of the separated morphotypes were examined in both NB2 and in water. There was no significant difference (*p* > 0.5; ANOVA) in the ability of the two morphotypes to survive in NB2 at either 37 or 4°C (**Figures 4A,B**). However, marked differences were observed in the survival of the two morphotypes incubated in water at either temperature (**Figures 4C,D**). The viable count of the short spiral forms fell below the limit of detection after 72 h at 37°C and 96 h at 4°C, whilst the filamentous forms remained detectable at 37°C and experienced only a modest fall in viability of 1.5 log_10_ over 96 h at 4°C.

### ATP Contents of Spiral, Filamentous, and Coccal Cell Types

**FIGURE 5:**
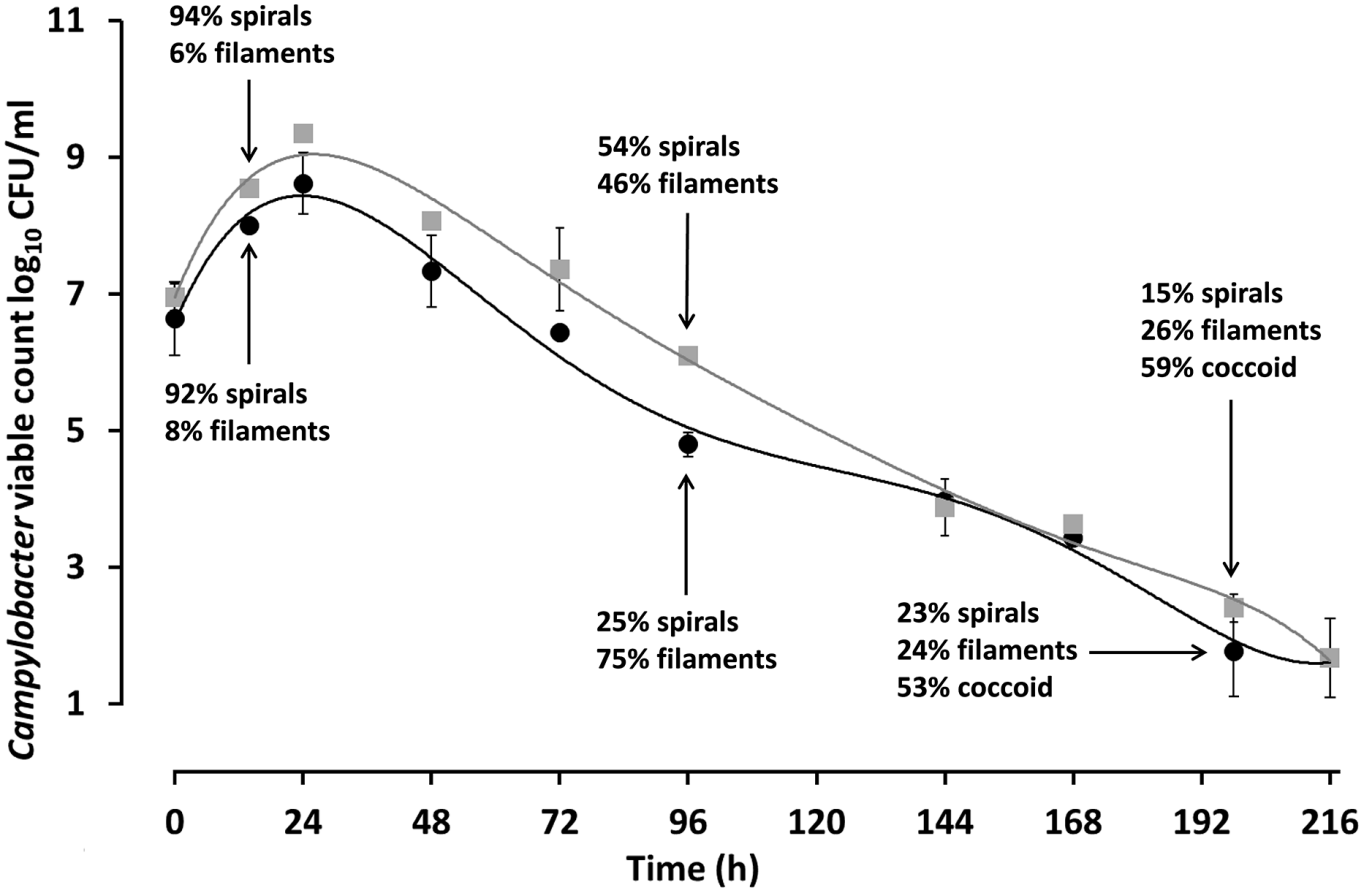
**Viable counts and microscopic enumeration of the cell morphotypes observed during microaerobic growth of *C. jejuni* PT14 and 12661.** *C. jejuni* PT14 (●) and 12661 (□) were cultured in MEM supplemented with 10 mM sodium pyruvate under microaerobic conditions at 37°C, from which samples were taken for microscopic examination and estimates of cell bound ATP. Error bars are ±SD for *n* = 3.

In order to study the ATP contents of spiral, filamentous and col types, *C. jejuni* 12661 (exaggerated filamentation phenotype) and *C. jejuni* PT14 (a strain that showed a typical filamentation phenotype) were selected. Viable and microscopic counts of microaerobic cultures at 37°C of *C. jejuni* 12661 and PT14 in MEM with sodium pyruvate were collected over 216 h to encompass exponential, stationary and decline phases of growth (**Figure 5**). Spiral cells harvested in mid-exponential phase at 14 h were serially diluted and the ATP contents of these cells estimated from luciferase/luciferin luminescence after lysis (**Table 2**). The ATP contents of the exponential phase spiral cells (0.99 and 1.7 fg ATP per CFU) were similar to earlier estimates for *C. jejuni* (Ng et al., 1985). Recovery and separation of filamentous and spiral forms in decline phase at 96 h enabled determination of the ATP contents of these cell populations before the appearance of coccal forms. Beyond 168 h the majority of the cells appeared coccal in these cultures, and estimates of viability required the plating of 0.2 ml culture volumes on multiple blood agar plates and/or recovery at endpoint dilution in broth cultures. Microscopic evaluation of 216 h cultures revealed that 61% of the *C. jejuni* 12661 cells had become coccal compared with 58% for *C. jejuni* PT14. After correction for the ATP contents of decline phase spiral and filamentous cells present in these cultures (**Figure 5**), estimates of the ATP contents of the dominant coccal cells were calculated (**Table 2**). The low or undetectable ATP contents of the coccal cells would support the conclusion that they are no longer viable, and that the viable campylobacters recovered from these cultures represent the remaining spiral and filamentous forms.

**Table 2:** Intracellular ATP content of *C. jejuni* morphotypes at different phases of growth.

Cell Forms*	*C. jejuni* 12661	*C. jejuni* PT14
	ATP (fg/cell)^†^	ATP (fg/cell)^†^
Exponential phase spirals (14 h)	1.7 ± 0.35	0.99 ± 0.12
Decline phase spirals (96 h)	1.19 ± 0.20	0.84 ± 0.14
Decline phase filaments (96 h)	17.4 ± 2.2	2.66 ± 0.54
Decline phase coccoid (196 h)	0.01 ± 0.005	ND
Decline phase coccoid (216 h)	0.01 ± 0.004	ND

*Cell morphotypes observed at different time points (h) in microaerobic culture of MEM with pyruvate. ^†^±SD *n* = 3. ND, not detectable.

## Discussion

Growth curves and microscopic examination of broth grown cultures confirmed that filamentous morphotypes were formed upon entry to stationary phase. Vital staining revealed interesting structural features of the filamentous cells but also that this method may not be suitable for distinguishing live *Campylobacter* cells from dead ones. The staining patterns observed suggest growth and septa formation occur within the filaments, and that this leads to differential dye permeability preventing dye migration between cells. Moreover, the interspersed pattern would suggest cell division may be taking place at the predefined cell poles within the filament suggesting at least some of the component cells were viable. Cameron et al. (2012) observed irregular patterns of cells remaining unstained by PI within *Campylobacter* filaments formed in response hyperosmotic stress. This prompted the authors to investigate septa formation using Vanco-FL stain that binds D-Ala-D-Ala moieties of peptidoglycan indicative of septa and/or sites of new peptidoglycan synthesis. The Vanco-FL stain produced a punctuated staining pattern in hyperosmotic-induced filaments that did not co-localize with PI. This observation is also indicative of internal septa formation, and as the authors conclude phenotypic differences between cells that compose the filament.

Quorum sensing bacteria produce and release auto inducers that increase in concentration as a function of cell density leading to an alteration in gene expression (Miller and Bassler, 2001) and in some cases filamentation (Allison et al., 1992). As no change in the time taken to form filaments occurred when pre-used medium was inoculated with a fresh culture, it seems unlikely that quorum sensing was involved in the process. The relatively proficient growth of campylobacters in the pre-used medium was unexpected, as nutrient depletion on entry to stationary phase was suggested as a possible trigger for filamentation. This raises questions as to what actually triggers entry to stationary and decline phases in *Campylobacter* broth cultures. This is not well understood in other bacteria and probably depends on a combination of factors including the species of bacteria and growth medium. For *Salmonella typhimurium* and *Escherichia coli* it has been shown that increased levels of acetate (Wilson et al., 2003) or carbon starvation (Sezonov et al., 2007) are associated with the cessation of growth. The transition to stationary phase for *Campylobacter* is highly dynamic with a switch from acetate production to utilization together with a peak in motility and numerous gene expression changes (Wright et al., 2009). In addition, the bacteria do not appear to exhibit enhanced stress resistance in stationary phase, unlike many other bacteria, which is consistent with the absence of RpoS homologues (Kelly et al., 2001). Despite the absence of RpoS, campylobacters retain a stringent response that assists survival in the stationary phase of many bacterial species. Mutation of the *spoT* gene results in aberrant cell morphologies and early coccoid cell formation in stationary phase cultures (Gaynor et al., 2005).

As a microaerophile, *Campylobacter* is particularly sensitive to the presence of free radicals and may suffer oxidative stress when grown in broth media despite being supplied with a reduced oxygen atmosphere (John et al., 2011). Strictly anaerobic conditions in the presence of alternative electron acceptors nitrate or fumarate have also been shown to induce filamentation in *Campylobacter* indicating an oxygen requirement for DNA synthesis (Sellars et al., 2002). The presence of starch in *Campylobacter* culture media acts as an antioxidant and affords a degree of protection against oxidative stress caused by free radicles that can accumulate during exponential growth (Mehlman and Romero, 1982). The provision of starch in the culture media did not influence the timing or degree of filamentation, which suggests that the accumulation of reactive oxygen species (ROS) in the growth medium is not a predisposing factor to the appearance of the filamentation morphotype. However, this does not rule out a role for oxidative stress at a cellular level. Endogenous ROS produced as a consequence of cellular metabolism have been suggested to play a role as signaling molecules and effectors in the development of microbial multi-cellularity, including programed cell death (Čáp et al., 2012).

Campylobacters were unable increase in viable count in MEM, without a carbon source but some increase in cell size was observed. The addition of pyruvate to the MEM allowed growth but with the early onset of filamentation as compared with growth in nutrient rich medium. Nutritional differences clearly impact on the observed morphological changes but since pyruvate remained in excess during the growth period as a carbon and energy source, it is unlikely that filamentation is a response to carbon starvation in these experiments. Bacteria also require sufficient iron, phosphorous, sulfur, nitrogen, and other trace elements for growth and it is possible that one, or a combination of these, become quickly exhausted in minimal medium resulting in filamentation, compared to rich media. However, the response to nutrient limitation even within a well-mixed *Campylobacter* broth culture is not uniform, in most cultures filamentous types arise among spirals that continue to divide. This implies that once the growth rate has become limited due to nutritional availability, then the formation of the filaments is either a stochastic process or developmentally controlled to generate a subpopulation that are more able to survive nutritional depletion and/or environmental stresses. In the wider environment other limiting physiological factors may also trigger the filamentation response.

*Campylobacter jejuni* cultured in MEM with pyruvate exhibit an ability to retain a viable subpopulation through decline phase at 37°C under microaerobic conditions despite a fall in viable count from >8 log_10_ CFU/ml at the end of exponential phase (24 h in **Figure 5**) to 3 log_10_ CFU/ml at 168 h. We have measured the ATP contents of cell morphotypes recovered from the decline phases of these cultures to demonstrate significant increases in the cellular ATP contents of the filamentous types as compared with spiral forms, sampled in either exponential phase, or separated from filaments in decline phase. These increases may be accounted for because the filaments appear to consist of multiple cells, joined in an ordered conglomerate. Consistent with this view, is the observation that the *C. jejuni* strain 12661 produces notably longer filaments and has a higher ATP content (17.4 fg ATP/CFU) than *C. jejuni* PT14 (2.66 fg ATP/CFU). However, we have noted interspersed staining patterns and cell division within filaments, which could represent cells with greater metabolic activity within a single filament. In the later stages of these cultures (>168 h) the majority of the cells become coccoid. ATP content estimates of coccal cells were either extremely low or non-detectable, suggesting they are not viable as concluded in previous reports (Moran and Upton, 1986; Boucher et al., 1994).

Differences in the ability of *Campylobacter* isolates to survive in microcosm waters have been documented but without reference to the formation of filaments (Buswell et al., 1998). The increased ability of the filamentous morphotype to survive in water compared to the short spiral form suggests that further research is necessary to assess the impact this may have on the transmission of the campylobacters from the environment to farm animals and on the safety of post-process foods. Based on these observations caution is advised when applying mathematical models that predict the survival *Campylobacter*, but do not take into account that changes in cell morphology and physiology that can increase their probability of survival. The presence of multicellular filaments may also lead to underestimates of viable cell numbers in cultures, since a single filament can form a single colony despite a multicellular origin, and filaments can exhibit differing refraction properties to non-dispersed cell suspensions that can make the interpretation of optical density measurements problematic (Wright et al., 2009).

Rapid filamentation was observed in MEM with pyruvate, in all the *C. jejuni* tested, and in all but one of the *C. coli* strains tested regardless of the strains being of either poultry or human origin. The response appears widespread amongst the two species. The minimal medium employed in these experiments did not support any observable growth of the single *C. coli* strain that did not form filaments, it could therefore not be concluded that this strain lacked the capacity to form filaments.

Laboratory based experiments of protein expression and metabolism of *Campylobacter* demand the use of broth grown cultures to control the phase of growth. Cultures are often harvested in late exponential phase to maximize cell yields, which can contain filamentous forms with potentially different characteristics to exponentially grown short spiral forms. Whilst it has long been recognized that growth *in vitro* cannot necessarily be compared with growth *in vivo*, the observation that *in vitro* grown cells harvested late in the growth cycle show such radically different morphology and survival ability, to cells in exponential phase, requires consideration in the context of experimental design and the methods to be adopted for environmental surveys. The filamentation of *Campylobacter* strains in laboratory broth culture appears to be a common occurrence. Whether filamentation occurs in all environments inhabited by campylobacters, for example in the intestines of animals and birds, is not known but the presence of filaments in biofilms indicates that this should be further investigated. Similarly there are no reports of filamentous forms observed from environments such as water courses, or from chilled meat, where campylobacters survive but have limited growth potential.

## Author Contributions

NG performed the experimental analyses. NG, PC, and IC designed the experiments, interpreted the data, and wrote the manuscript.

## Conflict of Interest Statement

The authors declare that the research was conducted in the absence of any commercial or financial relationships that could be construed as a potential conflict of interest.
